# FEELING YOUNGER, BEING YOUNGER: ASSOCIATIONS BETWEEN BIOLOGICAL AGE AND SUBJECTIVE AGE IN OLDER ADULTS

**DOI:** 10.1093/geroni/igz038.2291

**Published:** 2019-11-08

**Authors:** Johanna Drewelies, Ilja Demuth, Sandra Duezel, Gizem Hueluer, Lars Bertram, Elisabeth Steinhagen-Thiessen, Denis Gerstorf

**Affiliations:** 1 Humboldt University Berlin, Berlin, Germany, Germany; 2 Charité – Universitätsmedizin, Berlin, Berlin, Germany; 3 Max Planck Institute for Human Development, Berlin, Berlin, Germany; 4 University of Zurich, Zurich, Zurich, Switzerland; 5 University of Lübeck, Lübeck, Schleswig-Holstein, Germany; 6 Humboldt University, Berlin, Berlin, Germany

## Abstract

Subjective age has been shown to be a strong predictor of both subjective and objective health outcomes. However, little is known about the extent to which individuals’ subjective age is related to one’s biological age or not. In our study, we examine how subjective age relates to biological age—a comprehensive multi-indicator biomarker algorithm aggregating information of metabolic, cardiovascular, inflammatory, lung, and kidney functioning. We used data from 996 older adults from the Berlin Aging Study II (mean age = 68.40 years, range 60 to 85, 52% women) who provided information about chronological age, biological age, and subjective age. Multiple regression analyses revealed that subjective age was associated with biological age among older women with and without controls for age, education, and physician-observed comorbidity, but not older men. Our findings suggest that subjective age might provide unique insights into how biological age differs across adulthood and contributes to overall health.

